# Preoperative Motor and Sensory Dysfunction as Predictors of Permanent Neurological Deficit in Spinal Tuberculosis: A Retrospective Cohort Study

**DOI:** 10.7759/cureus.108105

**Published:** 2026-05-01

**Authors:** Thitikan Wangapakul, Alejandro Becerril-Mejía, Ambar Elizabeth Riley Moguel, Janeth N. Nuñez-Lupaca, Rodrigo Jesus Flores Palacios, Jesús Oswaldo Díaz Lagunas, Xavier Wong Achi

**Affiliations:** 1 Neurological Surgery, Yala Hospital, Yala, THA; 2 Neurological Surgery, Instituto Nacional de Neurologia y Neurocirugía, Mexico City, MEX; 3 Neurosurgery, Instituto de Seguridad y Servicios Sociales de los Trabajadores del Estado (ISSSTE), Mexico City, MEX; 4 Neurosurgery, Universidad Nacional Jorge Basadre Grohmann, Tacna, PER; 5 Faculty of Medicine, Universidad Nacional Jorge Basadre Grohmann, Tacna, PER; 6 Functional Neurosurgery, Instituto Nacional de Neurología y Neurocirugía Manuel Velasco Suárez, National Autonomous University of Mexico, Mexico City, MEX; 7 Neurological Surgery, Instituto Nacional de Neurología y Neurocirugía, Mexico City, MEX

**Keywords:** neurological deficit, pott's disease, prevalence ratio, spinal tuberculosis, spine surgery

## Abstract

Background: Spinal tuberculosis (TB) remains a leading cause of permanent neurological deficit in endemic regions. Identifying preoperative factors associated with poor neurological outcomes could inform surgical decision-making, but high-quality predictor data are limited.

Methods: We retrospectively analyzed 27 consecutive adult patients who underwent surgical treatment for spinal TB at a tertiary neurosurgical center between January 2018 and December 2022. Permanent neurological deficit was defined as persistent motor weakness (Medical Research Council [MRC] grade ≤4) or sensory impairment at a minimum of 12 months postoperatively. Univariable associations were assessed using chi-square or Fisher's exact tests, with prevalence ratios (PR) and 95% confidence intervals (CIs).

Results: Fifteen patients (55.6%) developed permanent neurological deficit. Two preoperative variables were significantly associated with poor outcomes: severe motor dysfunction (MRC 0-3) (PR 3.82, 95% CI 1.08-13.58; Fisher's exact test, p = 0.007) and diminished or absent sensation (PR 2.55, 95% CI 1.08-6.03; Fisher's exact test, p = 0.021). Cervical-thoracic involvement showed a non-significant trend toward worse outcomes (PR 2.20, 95% CI 0.93-5.18; Fisher's exact test, p = 0.057). Age, sex, comorbidities, sphincter dysfunction, number of vertebral levels affected, and radiological features including epidural compression were not significantly associated with permanent deficit.

Conclusions: Preoperative motor and sensory status are the strongest available predictors of permanent neurological deficit in patients undergoing surgery for spinal TB. These findings support early identification and prioritization of patients with severe preoperative neurological compromise. Larger multicenter studies are needed to develop and externally validate quantitative risk prediction tools.

## Introduction

Spinal tuberculosis (TB), also known as Pott disease, accounts for approximately half of all cases of musculoskeletal TB and remains an important cause of neurological disability worldwide [1,2]. Despite advances in antimycobacterial therapy and surgical techniques, permanent neurological deficits affect a substantial proportion of patients, reported in 23% to 76% of surgical series [3,4]. Once established, these deficits have a lasting impact on quality of life and functional independence, particularly in the working-age populations most often affected.

The pathophysiology of neurological injury in spinal TB is multifactorial. Mechanical compression from epidural granulation tissue, cold abscesses, vertebral collapse, and direct inflammatory effects on neural tissue may all contribute [5]. Although early surgical intervention is generally favored when neurological compromise is present, controversy persists regarding optimal timing, patient selection, and the preoperative findings that most reliably predict long-term outcome [6,7].

Previous studies examining outcome predictors in spinal TB have been limited by small sample sizes, heterogeneous patient populations, inconsistent outcome definitions, and limited use of standardized neurological scales [8,9]. As a result, surgeons in endemic regions often rely on clinical judgment and experience rather than on evidence-based criteria when counseling patients about prognosis.

The purpose of this study was to identify preoperative clinical and radiological factors associated with permanent neurological deficit in a consecutive cohort of patients undergoing surgical treatment for spinal TB. This analysis is prognostic in nature and is not intended to inform the diagnosis of spinal TB, which is established through microbiological, histopathological, and imaging criteria, as described separately in the Materials and Methods section.

## Materials and methods

Study design and setting

We conducted a retrospective cohort study of all consecutive adult patients who underwent surgical treatment for spinal TB at a tertiary neurosurgical center between January 2018 and December 2022. The study was approved by the institutional review board, which waived the requirement for informed consent given the retrospective and anonymized nature of the analysis. All procedures were conducted in accordance with the Declaration of Helsinki.

Patient selection

Eligible patients were aged 18 years or older, had radiologically and microbiologically and/or histopathologically confirmed spinal TB, were treated surgically as primary management, and had a complete preoperative neurological examination and a minimum of 12 months of clinical follow-up. Patients were excluded for concurrent spinal pathology (malignancy or non-mycobacterial infection), prior spinal surgery at the affected levels, incomplete medical records, or loss to follow-up before 12 months. The diagnosis of spinal TB required at least two of the following: characteristic MRI findings, positive microbiological confirmation (acid-fast bacilli smear, GeneXpert, or culture), histopathological evidence of granulomatous inflammation, and clinical response to antituberculous therapy.

Variables and outcome

Data extracted from medical records included demographics, symptom duration, preoperative motor function (Medical Research Council [MRC] scale), sensory examination, sphincter function, radiological features (spinal level, number of segments involved, epidural compression, abscess formation, vertebral destruction), and surgical details. Spinal level was categorized as cervical-thoracic versus lumbar-sacral; purely cervical involvement was grouped together with thoracic involvement because both regions expose the spinal cord proper (as opposed to cauda equina) and because the number of isolated cervical cases in the source records was too small to support a three-level stratification. The primary outcome was permanent neurological deficit, defined as persistent motor weakness (MRC grade ≤4) or sensory impairment at the final follow-up visit (minimum of 12 months postoperatively). The MRC ≤4 cutoff was selected because grade 4 strength, while permitting active movement against resistance, represents a clinically meaningful functional limitation in this predominantly working-age population.

Statistical analysis

Continuous variables are reported as mean ± standard deviation, while categorical variables are reported as frequencies and percentages. Univariable comparisons between patients with and without permanent deficit were performed using the chi-square or Fisher's exact test, as appropriate. Prevalence ratios (PR) with 95% confidence intervals (CIs) were calculated for each candidate predictor. Given the limited number of outcome events (n = 15), no multivariable model is presented in this report; the analysis is intentionally restricted to univariable associations to avoid overfitting. A p-value of less than 0.05 was considered statistically significant. Analyses were performed using IBM SPSS Statistics for Windows, Version 28.0 (Released 2021; IBM Corp., Armonk, New York, USA) and Python 3.9.

## Results

Patient characteristics

Twenty-seven patients met the inclusion criteria. The mean age was 42.7 ± 14.8 years, and 15 patients (55.6%) were male. Most patients (88.9%) had at least one comorbidity. The median symptom duration before presentation was 90 days (interquartile range, 30-365), with 11 patients (40.7%) presenting acutely (<3 months). Cervical-thoracic involvement was the most common pattern (15 patients, 55.6%), followed by lumbar-sacral (8 patients, 29.6%) and multi-segment disease (3 patients, 11.1%). Preoperative neurological characteristics are summarized in Table 1.

**Table 1:** Baseline characteristics of the cohort (n = 27). MRC: Medical Research Council; SD: standard deviation.

Characteristics	Value
Age, years (mean ± SD)	42.7 ± 14.8
Male sex, n (%)	15 (55.6)
Comorbidities, n (%)	24 (88.9)
Acute presentation (<3 months), n (%)	11 (40.7)
Severe motor dysfunction (MRC 0–3), n (%)	17 (63.0)
Diminished/absent sensation, n (%)	14 (51.9)
Sphincter dysfunction, n (%)	8 (29.6)
Cervical–thoracic involvement, n (%)	15 (55.6)
Multi-level involvement (≥2 levels), n (%)	14 (51.9)
Severe vertebral destruction (>50%), n (%)	12 (44.4)
Epidural compression, n (%)	21 (77.8)

Primary outcome and predictor analysis

Fifteen patients (55.6%, 95% CI 35.3%-74.5%) developed a permanent neurological deficit at final follow-up. Among these, 12 (80.0%) had persistent motor deficits, 8 (53.3%) had sensory deficits, and 5 (33.3%) had combined motor and sensory impairment.

**Figure 1:**
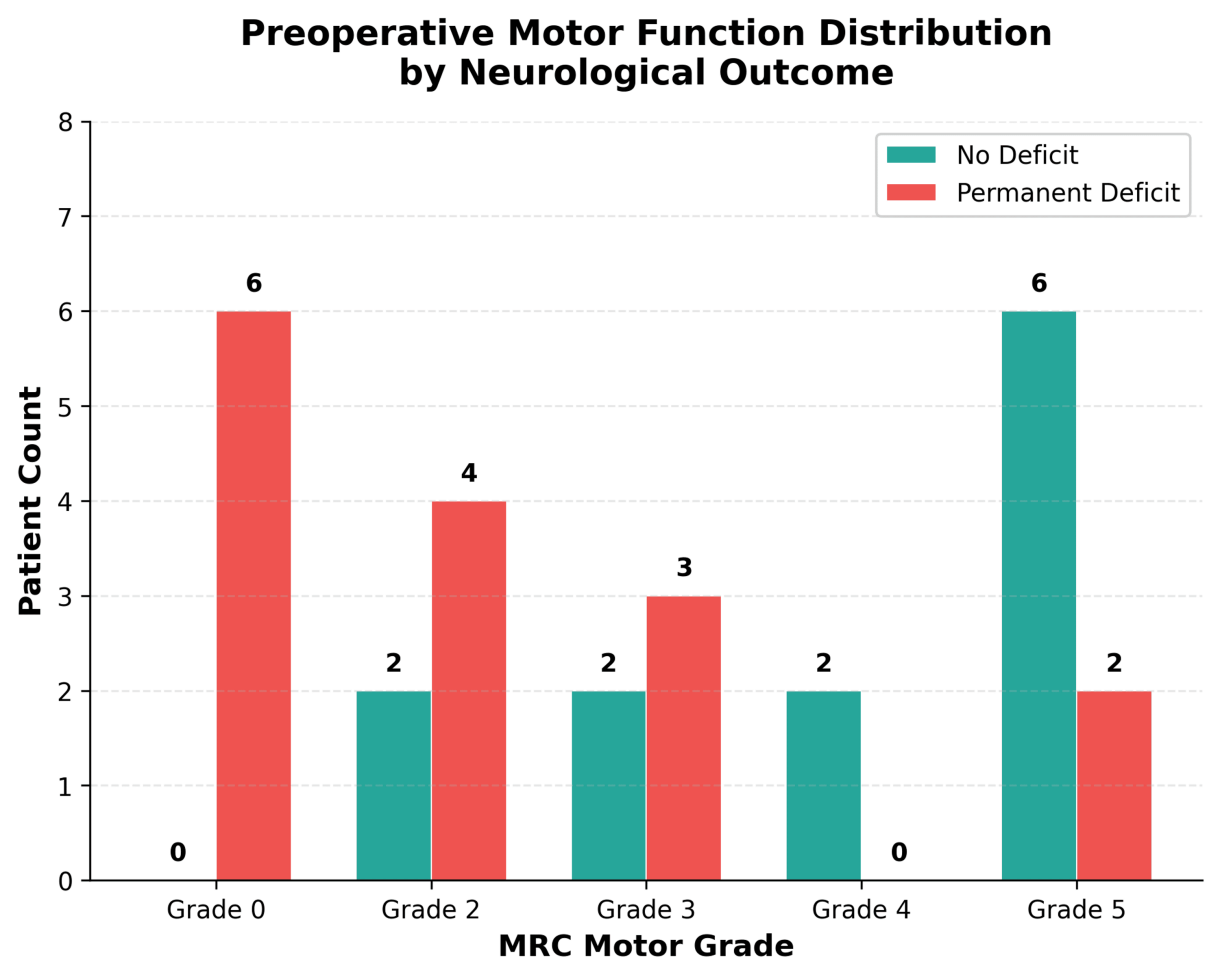
Distribution of preoperative motor function (MRC scale) by neurological outcome. Patients presenting with severe motor dysfunction (grades 0–3) were substantially more likely to develop permanent deficit. MRC: Medical Research Council.

Univariable analysis identified two preoperative variables that were significantly associated with permanent neurological deficit: severe motor dysfunction (MRC 0-3) (PR 3.82, 95% CI 1.08-13.58; Fisher's exact test, p = 0.007) and diminished or absent sensation (PR 2.55, 95% CI 1.08-6.03; Fisher's exact test, p = 0.021). The distribution of preoperative motor grades by outcome is shown in Figure 1, illustrating the marked concentration of permanent deficits among patients presenting with MRC grades 0-3. Cervical-thoracic involvement (PR 2.20, 95% CI 0.93-5.18; Fisher's exact test, p = 0.057) and acute symptom onset of less than three months (PR 1.89, 95% CI 0.81-4.42; Fisher's exact test, p = 0.130) both showed clinically relevant trends but did not reach statistical significance; their 95% CIs included the null value of 1.0. Sphincter dysfunction, although clinically relevant, was also not statistically associated with permanent deficit in this cohort (PR 1.80, 95% CI 0.71-4.59; p = 0.163), likely reflecting limited power given the small number of patients with bladder or bowel involvement (n = 8).

**Figure 2:**
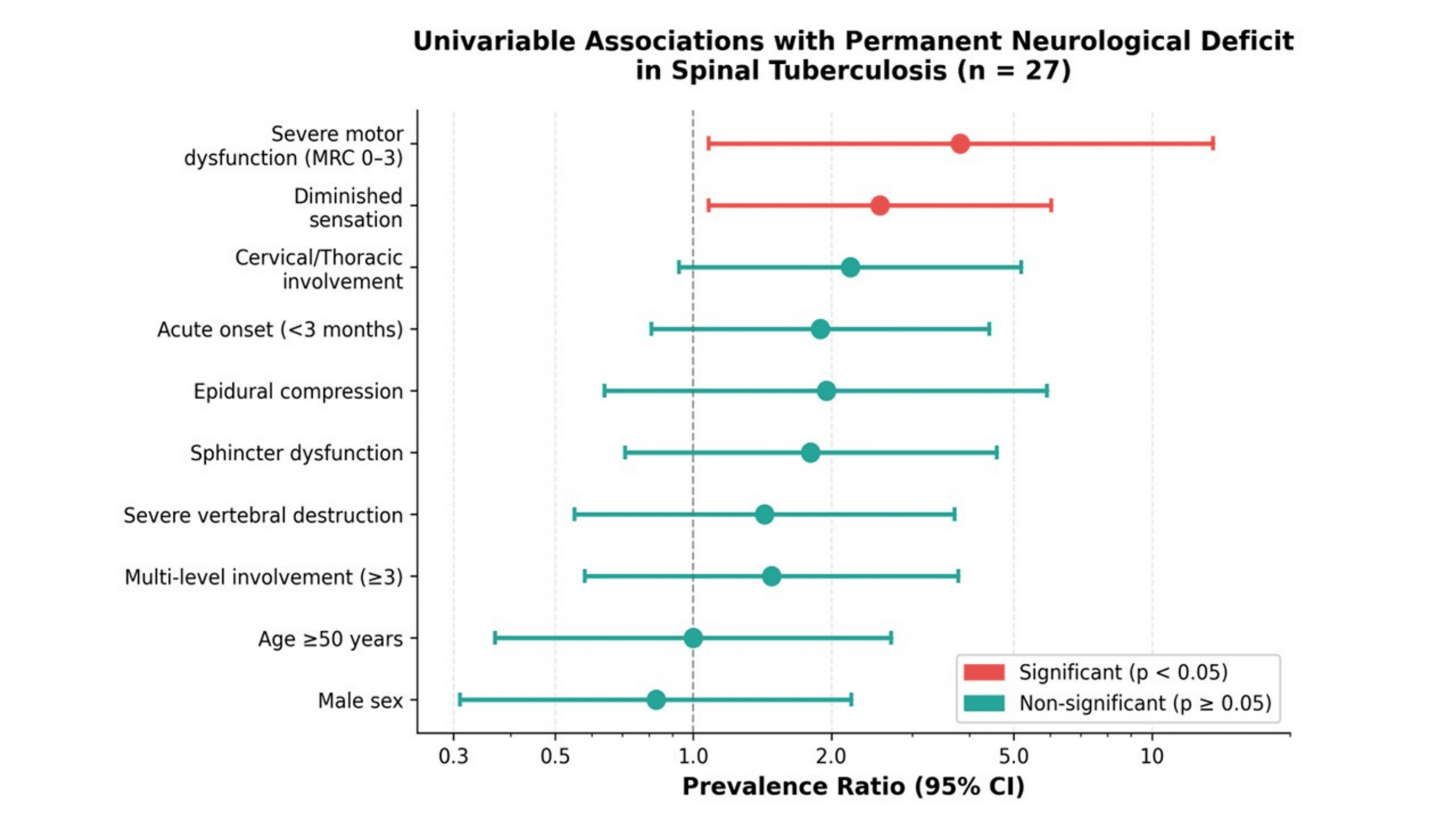
Forest plot of preoperative factors and their association with permanent neurological deficit (n = 27). Statistically significant predictors (p < 0.05) are shown in red. Severe motor dysfunction and diminished preoperative sensation were the only factors with CIs excluding 1.0. CI: confidence interval.

Demographics, comorbidities, number of affected levels, vertebral destruction, and presence of epidural compression were not significantly associated with the primary outcome (Table 2). A forest plot summarizing the prevalence ratios and 95% CI for all candidate predictors is presented in Figure 2.

**Table 2:** Univariable analysis of preoperative factors associated with permanent neurological deficit. CI: confidence interval; MRC: Medical Research Council; PR: prevalence ratio. p-values denote statistical significance (p<0.05).

Variables	No deficit (n=12)	Deficit (n=15)	PR (95% CI)	p-value
Severe motor dysfunction (MRC 0–3)	4 (33.3)	13 (86.7)	3.82 (1.08–13.58)	0.007
Diminished sensation	3 (25.0)	11 (73.3)	2.55 (1.08–6.03)	0.021
Cervical–thoracic involvement	4 (33.3)	11 (73.3)	2.20 (0.93–5.18)	0.057
Acute onset (<3 months)	5 (41.7)	11 (73.3)	1.89 (0.81–4.42)	0.130
Sphincter dysfunction	2 (16.7)	6 (40.0)	1.80 (0.71–4.59)	0.163
Epidural compression	8 (66.7)	13 (86.7)	1.95 (0.64–5.90)	0.357
Multi-level involvement (≥3 segments)	6 (50.0)	10 (66.7)	1.48 (0.58–3.78)	0.333
Severe vertebral destruction	4 (33.3)	8 (53.3)	1.43 (0.55–3.71)	0.259
Age ≥50 years	4 (33.3)	5 (33.3)	1.00 (0.37–2.70)	1.000
Male sex	7 (58.3)	8 (53.3)	0.83 (0.31–2.21)	0.766

## Discussion

In this consecutive single-center cohort of 27 patients undergoing surgical treatment for spinal TB, more than half developed a permanent neurological deficit at one-year follow-up. The two most reliable preoperative predictors of poor outcome were severe motor dysfunction (MRC grade 0-3) and diminished or absent sensation, both clinically straightforward findings that can be assessed at the bedside without specialized imaging or laboratory testing.

These findings are consistent with prior reports identifying preoperative neurological status as the dominant prognostic factor in spinal TB [8,10]. The magnitude of the associations observed here (a nearly fourfold increase in the prevalence of permanent deficit among patients with severe motor dysfunction) is clinically meaningful and supports the longstanding clinical principle that the depth of preoperative neurological compromise is the strongest single signal of likely outcome. Notably, structural radiological findings (number of vertebral levels involved, severity of vertebral destruction, and the presence of epidural compression) did not reach statistical significance in this cohort. While this may reflect limited statistical power, it also suggests that the functional neurological examination conveys prognostic information that is not fully captured by static imaging features.

Acute symptom onset (<3 months) and cervical-thoracic involvement both showed point estimates suggestive of increased risk (PR 1.89 and 2.20, respectively) but did not reach statistical significance. We interpret these non-significant trends with caution: the CIs were wide and included the null, reflecting the limited sample size. From a pathophysiological perspective, rapid clinical deterioration may be a marker of aggressive disease (greater inflammatory burden, more virulent infection, and faster cord compromise) rather than an independent causal mechanism; similarly, higher levels of spinal involvement expose neural tissue with less reserve and more vulnerability to compressive injury. Both hypotheses warrant confirmation in larger cohorts where multivariable adjustment is feasible.

Sphincter dysfunction, which reflects bladder and/or bowel involvement, is widely regarded in the spinal literature as one of the most important clinical predictors of neurological recovery. In acute cauda equina syndrome and other compressive myelopathies, the presence of bladder dysfunction is considered a surgical emergency and is independently associated with reduced long-term recovery when decompression is delayed [11,12]. In Pott disease specifically, case series have shown that patients with complete paraplegia and neurogenic bladder often retain vesico-sphincteric incontinence even after surgical decompression and prolonged rehabilitation [13]. In our cohort, sphincter dysfunction showed a point estimate consistent with increased risk of permanent deficit (PR 1.80, 95% CI 0.71-4.59; Fisher's exact test, p = 0.163); however, only eight patients presented with this finding, and the CI crossed unity. We therefore could not statistically confirm the importance suggested by prior literature, and we interpret this as a power limitation rather than evidence against the association. This variable warrants specific attention in larger cohorts, ideally with stratification by type (urinary urgency, retention, and bowel incontinence), severity, and timing of onset, which were not captured with sufficient granularity in our retrospective records. Future studies should consider urodynamic assessment as an adjunct to clinical examination [12].

Several limitations should be acknowledged. First, the sample size is small (n = 27, with 15 outcome events), which precludes meaningful multivariable modeling and limits statistical power for variables with smaller effect sizes; the associations reported here should therefore be interpreted as exploratory rather than validated predictors. Second, the 12-month follow-up window, while standard, may underestimate late functional recovery in patients with milder deficits. Third, the use of an MRC grade ≤4 as a threshold for permanent deficit is conservative; some patients meeting this definition retain substantial functional capacity. Fourth, spinal level was analyzed as cervical-thoracic versus lumbar-sacral; although cervical involvement has distinct biomechanical and prognostic considerations compared with thoracic disease, the small number of patients with isolated cervical involvement in this cohort did not support a separate analysis. Future multicenter studies with larger cervical subgroups should examine cervical disease as an independent stratum. Finally, granular information regarding the duration of preoperative neurological deficit (as opposed to total symptom duration), the severity and timing of sphincter dysfunction, and detailed surgical strategy or perioperative management was not consistently available in the source records and could not be incorporated into this analysis. Prospective multicenter studies with standardized outcome instruments and longer follow-up are needed to confirm these findings.

Despite these limitations, the consistency of the results with prior literature and the simplicity of the clinical predictors identified make this work practically actionable. Surgeons in endemic regions can use a brief preoperative motor and sensory examination to identify patients at substantially elevated risk of permanent deficit, supporting more informed surgical planning and patient counseling. Larger multicenter prospective studies, ideally with standardized outcome measures and external validation, are needed before quantitative risk prediction tools can be confidently developed and deployed in this population.

## Conclusions

In this exploratory single-center cohort of patients undergoing surgery for spinal TB, severe preoperative motor dysfunction (MRC grade 0-3) and diminished or absent sensation were the variables most strongly associated with a permanent neurological deficit. These findings, which should be interpreted as hypothesis-generating given the small sample, reinforce the central role of the bedside neurological examination in surgical decision-making and prognostic counseling for this population. Larger prospective multicenter studies are required to confirm these associations and to support the development and validation of quantitative risk prediction tools.
